# Open-Source Hardware May Address the Shortage in Medical Devices for Patients with Low-Income and Chronic Respiratory Diseases in Low-Resource Countries

**DOI:** 10.3390/jpm12091498

**Published:** 2022-09-13

**Authors:** Ramon Farré, David Gozal, Viet-Nhung Nguyen, Joshua M. Pearce, Anh Tuan Dinh-Xuan

**Affiliations:** 1 Unitat de Biofísica i Bioenginyeria, Facultat de Medicina i Ciències de la Salut, Universitat de Barcelona, 08036 Barcelona, Spain; 2 CIBER de Enfermedades Respiratorias, 28029 Madrid, Spain; 3 Institut Investigacions Biomèdiques August Pi Sunyer, 08036 Barcelona, Spain; 4 Department of Child Health, The University of Missouri School of Medicine, Columbia, MO 65201, USA; 5 National Tuberculosis Program, 463 Hoang Hoa Tham, Vinh Phu, Ba Dinh, Hanoi 118000, Vietnam; 6 Department of Electrical & Computer Engineering, Ivey Business School, Western University, London, ON N6A 5B9, Canada; 7 Service de Physiologie-Explorations Fonctionnelles, Hôpital Cochin, Assistance Publique-Hôpitaux de Paris (AP-HP), 75014 Paris, France

**Keywords:** medical devices, low-cost, low- and middle-income countries, flowmeters, mechanical ventilator, CPAP device, Arduino, 3D printer, open-source hardware

## Abstract

Respiratory diseases pose an increasing socio-economic burden worldwide given their high prevalence and their elevated morbidity and mortality. Medical devices play an important role in managing acute and chronic respiratory failure, including diagnosis, monitoring, and providing artificial ventilation. Current commercially available respiratory devices are very effective but, given their cost, are unaffordable for most patients in low- and middle-income countries (LMICs). Herein, we focus on a relatively new design option—the open-source hardware approach—that, if implemented, will contribute to providing low-cost respiratory medical devices for many patients in LMICs, particularly those without full medical insurance coverage. Open source reflects a set of approaches to conceive and distribute the comprehensive technical information required for building devices. The open-source approach enables free and unrestricted use of the know-how to replicate and manufacture the device or modify its design for improvements or adaptation to different clinical settings or personalized treatments. We describe recent examples of open-source devices for diagnosis/monitoring (measuring inspiratory/expiratory pressures or flow and volume in mechanical ventilators) and for therapy (non-invasive ventilators for adults and continuous positive airway pressure support for infants) that enable building simple, low-cost (hence, affordable), and high-performance solutions for patients in LMICs. Finally, we argue that the common practice of approving clinical trials by the local hospital ethics board can be expanded to ensure patient safety by reviewing, inspecting, and approving open hardware for medical application to maximize the innovation and deployment rate of medical technologies.

## 1. Introduction

Chronic and acute respiratory diseases—including chronic obstructive pulmonary disease, fibrosis, pneumonia, and acute respiratory distress syndrome—have been and continue to be an important healthcare concern worldwide [1]. Moreover, their prevalence and socio-economic burden are expected to grow owing to the progressive aging of the population and because some of the leading causes of chronic respiratory diseases—namely, air pollution, vaping, and tobacco smoking—are increasing, particularly in low- and middle-income countries (LMICs) [1]. The morbidity and mortality from respiratory diseases that are further enhanced by the surge of current (COVID-19) and future pandemics affecting the respiratory system along with the respiratory consequences of climate change and environmental pollution, particularly in at-risk subjects (patients with comorbidities, children, and the elderly), will only further accrue to the toll [2,3].

Vaccination strategies aimed at reducing the number of infected patients and the disease severity in case of infection, and the development of new drugs for improving treatment are two important tools for managing chronic and acute respiratory diseases. However, irrespective of such advances, a fundamental tool to address both acute and chronic respiratory illnesses consists of the use of a variety of medical devices. For example, a type of frequently used respiratory medical device is aimed at exploring the patient’s lung function for both diagnosing and monitoring the evolution of disease symptoms. Another type of device is used for treatment and primarily consists of mechanical ventilators that are required to support or replace the patients’ ability to breathe and, thus, maintain safe levels of O_2_ and CO_2_ in the different body tissues and organs.

Fortunately, intense engineering and clinical research in the last decades have made it possible first to invent and subsequently develop very sophisticated and effective medical devices for respiratory diseases. This excellent progress comes, however, with the drawback that, in practice, such advanced devices are not available to most of the populations in LMICs [4,5]. Indeed, most medical devices are produced and commercialized by companies in developed countries, with production costs; markup; and, thus, product prices that make the devices unaffordable to most individual patients and healthcare systems in LMICs. As a result, whereas almost all patients in developed countries and a small wealthy minority in LMICs have access to commercialized medical devices, billions of inhabitants in low-resource regions have scarce or no access to life-saving medical equipment such as mechanical ventilators.

Such widespread inequity in access to life-saving solutions prompts a uniquely important question aimed at addressing and resolving this global problem. In other words, what potential steps can be undertaken to eliminate or at least reduce this access inequality problem besides passively waiting under the wishful thinking that economic differences among countries will progressively disappear? Several strategies have been considered and implemented over the years. For example, philanthropic donations of commercially available medical devices can be a helpful solution for specific sites and needs. Unfortunately, philanthropy cannot be a universal solution owing to the very high costs involved (thus, inefficient use of donations), and also because the functioning time of donated medical devices to LMICs is short due to the lack of maintenance and repair capabilities [6]. Another solution that may help reduce inequalities in the access to medical devices is to promote their production and commercialization by local companies in LMICs since, in this case, the production and distribution costs can be markedly reduced [7]. Building so-called “frugal” medical devices can also help lower prices [8,9]. In frugal devices, the concept is to produce devices providing the most basic and necessary functions, thus, avoiding the costs corresponding to more complex or sophisticated functionalities that may be helpful for slightly improving the treatment, increasing profitability for the for-profit medical center, or for patient comfort, but are beyond the fundamental medical aim of the device. As a clear example, current advanced mechanical ventilators offer a wide variety of ventilation modes and monitoring options that are not essential for lifesaving and are not widely used in routine clinical practice. The alternative of fabricating less expensive devices by reducing non-fundamental functions or producing them in LMICs is very promising and should be actively promoted [10]. However, the costs incurred in any conventional industrial process and implementation, even in the case of not-for-profit companies in LMICs, may result in product prices still not sufficiently low to be affordable for many patients in low-resource regions. Indeed, a disproportionately large share of medical device companies’ revenues is slated toward expenses beyond those needed to manufacture their products [11]. Interestingly, a relatively new option, the open-source approach [12], may contribute to facilitate the provision of affordable respiratory medical devices for patients in LMICs.

## 2. The Open-Source Approach

Open source is defined as a way to design and distribute the technical information required for building devices such that anybody can freely and unrestrictedly use the know-how to replicate the device or modify the design for improvement or adaptation to different applications.

The free open-source concept was born several decades ago in the software industry as an alternative to proprietary software. The free and open-source software idea is that contributing engineers write and freely distribute routine codes, platforms, or applications so that other developers can eventually modify and incorporate them into their applications (e.g., Linux, Android) [13]. Open-source creates a gift economy [14] that encourages rapid innovation [15,16]. Free and open-source software development is now mature [17] and well documented to be a successful technical development method [18,19]. The open-source approach now dominates technical development in the software industry, with over 80% of the IOT (internet of things) market [20], over 84% of the global smartphone market [21], over 90% of the global cloud server market [22] (e.g., Facebook, Twitter, Yahoo, Google, and Amazon), over 90% of the Fortune Global 500 (e.g., Wal-Mart and McDonald’s) [23], and 100% of all supercomputers [24].

The concept of free open-source was subsequently expanded to hardware products, mainly thanks to the extension of two new technological developments [25,26]. One of them was the commercialization and widespread access to low-cost 3D printers through the RepRap project (Self-Replicating Rapid Prototypers, which literally fabricated their own components) [27,28,29]. Thus, open-source hardware developers contribute by building and freely distributing the files containing driving codes readable by conventional 3D printers [30]. Then, any individual can fabricate pieces made of different materials, which otherwise would require an expensive traditional process of design and fabrication by classical methods [31]. The use of low-cost desktop 3D printers [32] is now widely used not just for prototyping but to fabricate consumer goods at prices that are beneath those available in the conventional market [33]. The savings depend on the source of plastic used in the 3D printers: commercial filament (82%), commercial pellets (94%), recycled commercial pellets (97%), and self-recycled consumer plastics (98%) [34].

The other technological development that has expanded the open-source approach is the creation of standardized and simple electronic platforms allowing to read signals from sensors; carry out signal processing, including feedback control algorithms; and drive actuators in real-time (e.g., the Arduino electronic prototyping platform) [35]. These low-cost platforms do not necessarily have the highest performance offered by the most-novel, high-price options provided by the industry, but allow an enormous range of applications and are more than adequate for a vast array of scientific and medical applications [36,37,38]. Similarly, they also drive substantial cost savings. A recent survey of open hardware for science found the average open hardware tool provided 87% savings compared with equivalent or lesser proprietary tools, which increased slightly to 89% for those that used Arduino technology and even more to 92% for those that used RepRap-class 3D printing [39]. Combining both 3D printing and Arduino increased the savings that averaged 94% for free and open-source tools over commercial equivalents [39]. The combination of free open-source software and hardware approaches makes it possible for either expert engineers or individuals with basic technical knowledge to take advantage of the know-how provided by a vast community of developers worldwide [40]. The approach allows for a democratization of the production of high-value products not seen before [41].

Two relatively recent commercial novelties have made this all possible. First, the progressive cost reduction of electronic components, sensors, and actuators as a result of the globalization process in industrial chains. Second, the worldwide extension of e-commerce distribution channels (e.g., Amazon, eBay, Alibaba) eases the retail purchase of components. Consequently, it is currently possible for individuals or small labs to fabricate complex devices in a way that was not possible historically or even a few years ago. The fields of application of the open-source approach, which is considered a strategic and production-change paradigm, are ample in the current technology-based world, including industry, research, and medical devices [42].

The new abilities the open-source approach provides have been particularly useful during the COVID-19 pandemic. The conventional, centralized manufacturing of the proprietary approach to design and manufacturing failed to respond to the crisis in time and compounded existing inequalities in medical care, especially in LMICs [43,44]. There was a massive grassroots response to provide communities with access to necessary customized, locally produced equipment following a distributed manufacturing paradigm [45,46]. There has been a virtual explosion of open-source devices due to the supply disruptions and urgent need for medical hardware during the COVID-19 pandemic. Examples include the production of a wide range of personal protective equipment such as face shields [47], N95 respirator alternatives [48], heat-sterilizable masks [49], full-face masks [50,51], and testing supplies such as nasopharyngeal swabs [52,53] and infrared thermometers [54]. More complicated devices were also developed, including sterilization equipment [55,56,57] and open-source electronics for medical devices such as ventilators [58], among many others.

## 3. Low-Cost, Open-Source Devices for Respiratory Diseases

To better illustrate the potential of the open-source approach, this section provides a detailed analysis of some recent examples of low-cost diagnostic and therapeutic devices for chronic or acute respiratory diseases.

### 3.1. Device to Measure Maximal Inspiratory and Expiratory Pressures

**Figure 1:**
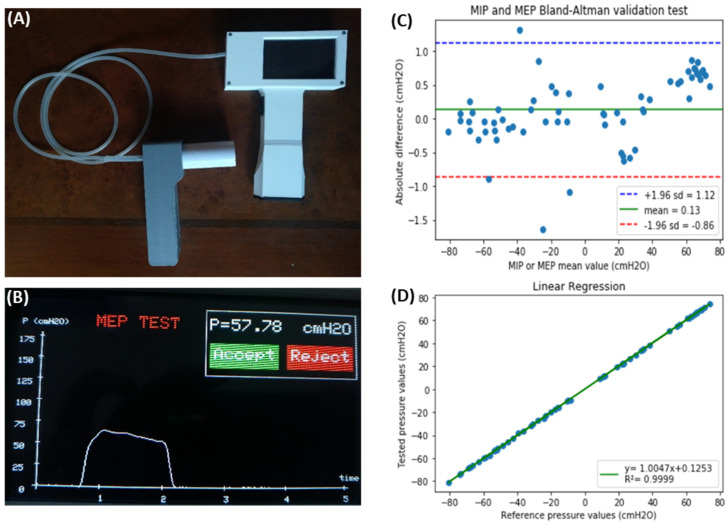
(**A**) Complete external view of the device showing the operator’s handheld block and the patient’s mouthpiece block, connected through a flexible silicone tube. (**B**) Example of one of the screens during device operation, showing the result of an MEP maneuver test (time course of expiratory pressure, MEP result, and the option to allow the user to accept or reject this specific maneuver). (**C**) Bland–Altman plot showing the difference between values measured by the prototype and by laboratory reference equipment, as a function of the measured values for both MIP (negative values) and MEP (positive values). The green line is the prototype bias, and the blue–red lines indicate the limits of agreement. (**D**) Linear regression of the values obtained with the developed device and the laboratory reference. Reprinted with permission from Ref. [59]. Creative Commons CC-BY license.

Measurement of the maximal inspiratory (MIP) and expiratory (MEP) pressures that a patient can exert allows for assessing the functional performance of his/her respiratory muscles. MIP and MEP are altered in very prevalent diseases such as chronic obstructive pulmonary disease (COPD), neuromuscular diseases (e.g., multiple sclerosis, muscular dystrophies), or chronic heart failure. Since measuring MIP and MEP is non-invasive, this technique helps in diagnosing and characterizing the disease and in monitoring the evolution of the patient’s disease status and response to therapy. Given that commercial devices for measuring MIP/MEP are expensive, a low-cost, open-source device has been recently proposed [59]. The MIP/MEP measurement technique is straightforward from both conceptual and technical viewpoints. It is based on recording pressures at a mouthpiece, computing the average of the highest pressures generated over a 1 s period of stable inspiratory/expiratory effort and providing the variability across values in subsequent maneuvers to select the maximum value from several representative efforts. The device (Figure 1A) was designed using simple, inexpensive, and easy-to-find components (most of them purchased from e-commerce sources). The setting consisted of a development board with an Arduino microcontroller, an LCD screen, a pressure transducer, a rechargeable 9 V battery block, a switch, a power supply base, and a customized enclosure produced by using any plastic-based 3D printer (most of which are open-source or derived from RepRap 3D printers). A measuring session starts by asking the user to select a MIP or MEP measurement to run; then, data acquisition begins immediately. After 5 s of pressure signal sampling, the device screen shows the corresponding pressure–time curve and the MIP/MEP values are computed according to conventional rules [59]. The device asks the user whether the maneuver should be accepted or rejected and whether a new maneuver should be carried out (Figure 1B). After repeated maneuvers, the device shows all data from previously accepted tests and informs on whether the quality control criterion to select the final result has been achieved [59]. Figure 1A shows that the device has two independent blocks connected through a 1 m length (3 mm ID) silicone tube. One of them is the handheld framework, used by the health technician, containing the electronics and digital display of the measurement process and results. The second block is a handheld mouthpiece support for a disposable patient’s mouthpiece. The cost of all the components was ≈ 80 €. Figure 1C,D show the results obtained when the low-cost, open-source device was evaluated by simultaneous comparison with a laboratory reference setting (Bland-Altman and linear regression plots, respectively). The average difference in MIP/MEP values between the low-cost device and the lab reference setting was only 0.13 cmH_2_O (limits of agreement from −0.86 to 1.12 cmH_2_O), which corresponds to ±1% accuracy. Accordingly, the developed device is suitable for performing MIP and MEP measurements within clinical ranges. The device design was published using the open-source hardware approach. Therefore, it can facilitate measuring MIP/MEP by readily available point-of-care devices for patient monitoring. Most importantly, it can make this respiratory function measurement tool affordable to users in LMICs.

### 3.2. Device to Measure the Tidal Volume Delivered by Mechanical Ventilators

**Figure 2:**
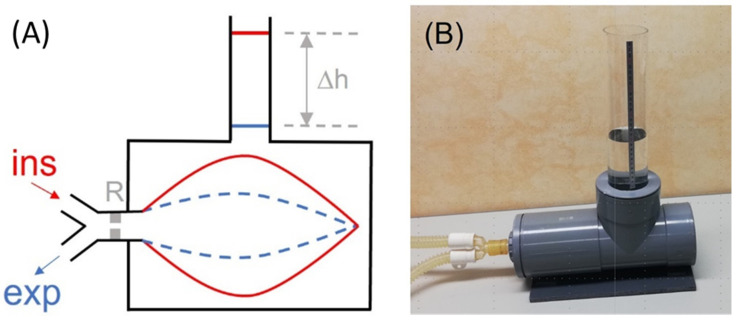
(**A**) Diagram of the method described for directly measuring the tidal volume (V_T_) delivered by a mechanical ventilator. A lung test, consisting of an orifice-type resistor (R) and a compliant bag enclosed in a water chamber open to the atmosphere through a vertical tube, is connected to the inspiratory and expiratory lines of the mechanical ventilator. The V_T_ introduced into the bag induces an increase in the height (∆h) of the water level in the tube, from end-expiration (blue) to end-inspiration (red). (**B**) Example of low-cost implementation of the measuring setting. The chamber was made with 15 cm diameter PVC drainpipe fittings. One of the cylinder bases was a screw cap to allow replacing the bag. The transparent vertical tube has an internal diameter of 7.4 cm (section: 43.01 cm^2^); hence, V_T_ (in mL) = 43.01 · h (in cm). Reprinted with permission from Ref. [60]. Creative Commons CC-BY license.

Tidal volume (V_T_), the volume of air achieved during inspiration, is one of the main parameters required during mechanical ventilation in patients with respiratory failure. Therefore, precise measurement of V_T_ is of great importance for both controlling blood gases and avoiding ventilator-induced lung injury. However, this measurement, usually carried out by the ventilator, is complex since it requires a series of corrections for oxygen concentration in the air, dead space of the ventilator circuit, and temperature and humidity conditions. As each of these three corrections may induce up to 10% variance in measured V_T_, periodic quality control testing is required, particularly in clinical settings where medical device maintenance could be suboptimal, for instance, in LMICs. To avoid the need for reference devices measuring V_T_, which are based on expensive gold standard sensors, a simple procedure has been recently described [60]. This procedure can be readily followed by clinical staff who are not experts in instrumentation techniques. Figure 2A shows a diagram of the rationale, which is based on measuring the volume of inspired air (V_T_) directly from water displacement. Interestingly, for users in LMICs, Figure 2B shows a low-cost implementation made using 15 cm diameter PVC tubing components that are widely available in hardware stores. Assuming the 1 mm resolution in the common ruler for assessing h in the setting in Figure 2B, the resolution in V_T_ measurement corresponds to 0.43% and 0.86% for maximum and typical V_T_ values of 1000 and 500 mL, respectively, being by far sufficient to detect any potential real-life errors when tidal volume is measured by mechanical ventilators.

### 3.3. Construction and Calibration of Accurate Pneumotachographs

**Figure 3:**
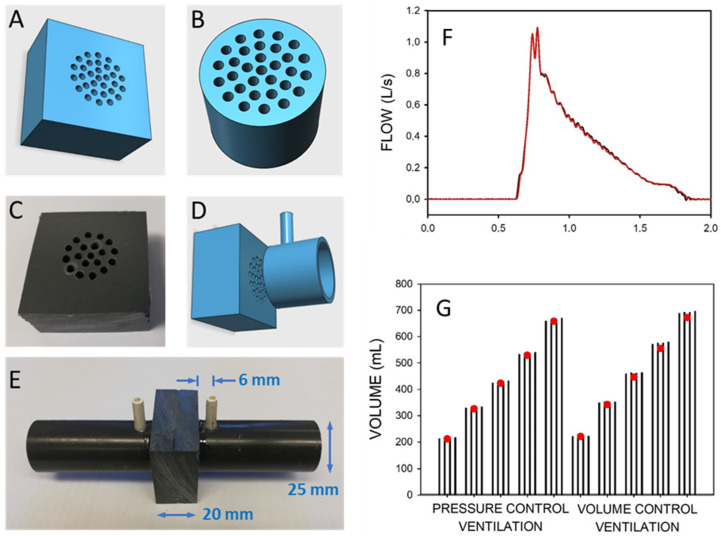
Low-cost pneumotachograph. (**A**,**B**) Diagrams of resistors for the pneumotachographs. (**B**,**C**) Photograph of the manually drilled resistor. (**C**,**D**) Diagram of resistor and (one side) standard PVC tube piece to assemble the pneumotach. (**E**) Photograph of the assembled pneumotachograph. (**F**) Example of the flow signals during pressure-controlled mechanical ventilation simultaneously measured by a reference pneumotachograph (red) and by the pneumotachograph constructed and calibrated by the low-cost procedures (black). (**G**) Volume measured during different magnitudes of pressure-control and volume-control mechanical ventilation of a patient model. Volume was simultaneously measured with the low-cost and the reference pneumotachograph (open circles). Each set of four bars corresponds to the volumes measured by the low-cost pneumotachograph using independent calibrations. Reprinted with permission from Ref. [61]. Creative Commons CC-BY license.

Pneumotachographs, which are the sensors measuring ventilation airflow (and volume by flow integration), are basic integral components required in mechanical ventilators. Given that standard pneumotachographs are expensive and require careful cleaning and maintenance, a recent publication has provided full details for low-cost and simple construction and calibration of robust pneumotachographs made by manual perforation of a plate with a domestic drill [61]. Their pressure–volume relationship is characterized by a quadratic equation with parameters that can be tailored by the number and diameter of the perforations (Figure 3A–E). The calibration parameters of the pneumotachographs can be measured through two maneuvers with a conventional resuscitation bag and by assessing the maneuver volumes with an inexpensive and straightforward water displacement setting. The performance of these simple, inexpensive pneumotachographs was assessed by comparison with a reference gold standard pneumotachograph in a bench test where conventional mechanical ventilation was applied to a simulated patient. As shown in Figure 3F,G, under realistic mechanical ventilation settings (pressure- and volume-control; 200−600 mL), the simple pneumotachographs were able to accurately measure inspiratory flow and tidal volume (V_T_ errors of 2.1% on average and <4% in the worst case). Therefore, these easy-to-reproduce pneumotachographs and the calibration method facilitate the low-cost and simple availability of pneumotachographs for accurately controlling mechanical ventilation in low-resource settings, either by incorporating them into the ventilators or as external measuring devices for quality control.

### 3.4. Pediatric Continuous Positive Airway Pressure (CPAP)

**Figure 4:**
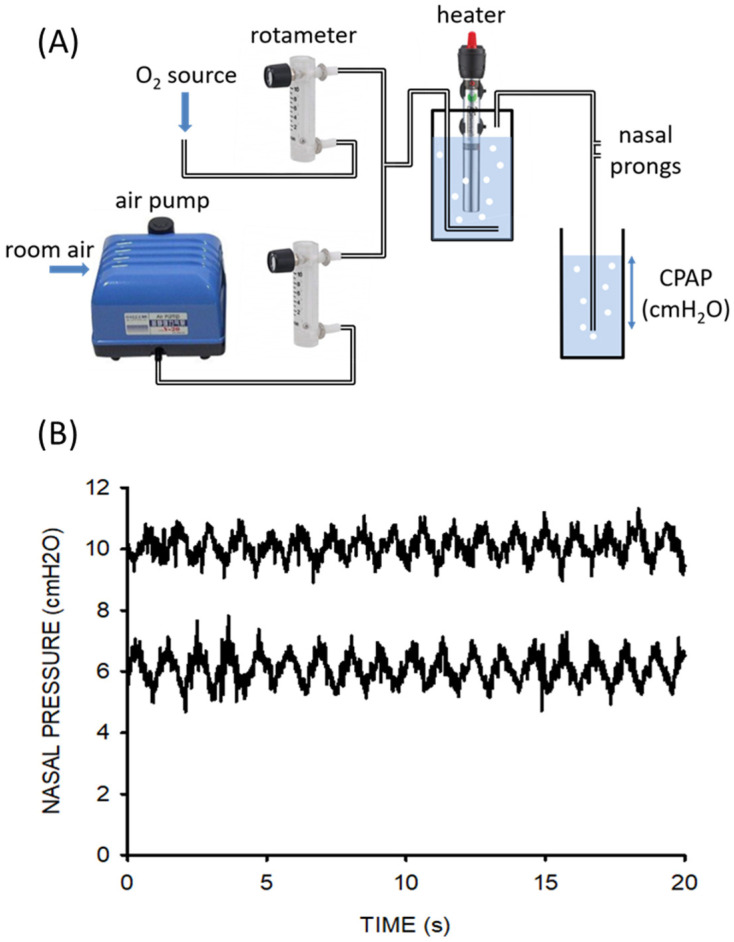
(**A**) Diagram of the CPAP setting, including its essential components: domestic aquarium air pump, rotameters, and domestic aquarium water heater/controller. (**B**) Nasal pressures actually applied at nasal prongs by the novel device for CPAP settings of 6 and 10 cm H_2_O. The simulated newborn infant was breathing with a tidal volume of 20 mL and frequency of 55 breaths/min while 8 L/min of humidified heated airflow circulated through the circuit. Reprinted with permission from Ref. [62]. Copyright by the American Thoracic Society.

The provision of therapy with continuous positive airway pressure (CPAP) has proven efficacy in the treatment of pneumonia in children, the leading cause of death in under-5-year-old patients in LMICs. Given the high cost of conventionally marketed CPAP devices, which are unaffordable for the majority of healthcare systems in low-resource regions, expanding access to potential CPAP treatment relies on the provision of simple and cheap devices for the application of this respiratory support therapy. Figure 4 shows in detail the example of a recent open-source proposal to facilitate the local construction of pediatric CPAP devices [62]. The setting (Figure 4A) is based on off-the-shelf, easy-to-purchase components (a pump and a heater/controller domestic aquarium, and two rotameters), which are assembled into a 3D printed enclosure. The total cost of the CPAP device was <100 €. When tested on the bench on a simulated patient with realistic breathing [62], the device was shown to provide CPAP support with precision and stability similar to commercial devices (Figure 4B).

### 3.5. Non-Invasive Pressure Support Ventilator

**Figure 5:**
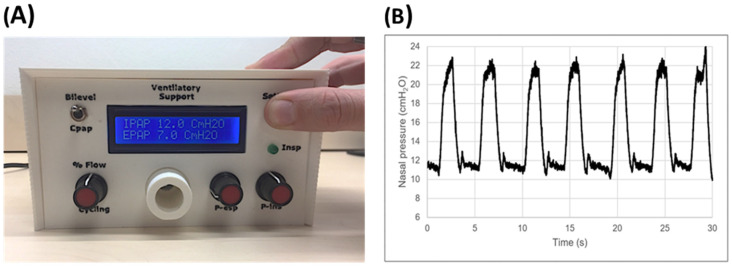
(**A**) Front view of the low-cost ventilator prototype. (**B**) Example of pressure signal recorded when the prototype ventilator supported a resistive–restrictive loaded breathing volunteer’s breathing. These are unfiltered raw data from the sensor within the ventilator. Reprinted with permission from Ref. [11]. Creative Commons CC-BY license.

**Figure 6:**
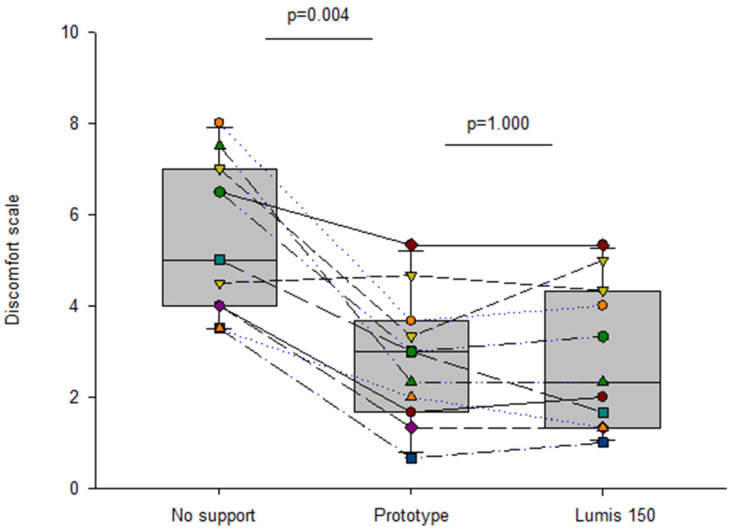
Discomfort scoring (Visual Analog Scale) in healthy volunteers subjected to obstructive–restrictive loaded breathing when unsupported and when supported by the prototype and Lumis 150 ventilators. Reprinted with permission from Ref. [11]. Creative Commons CC-BY license.

Current pricing of commercial mechanical ventilators in LMICs markedly restricts their availability, and consequently, a considerable number of patients with severe respiratory diseases cannot be adequately treated. To reduce the serious shortage of ventilators in low-resource regions, a recent simple, open-source, non-invasive bilevel pressure ventilator has been designed and evaluated in human subjects [11]. The ventilator (Figure 5A) was built using off-the-shelf materials available via e-commerce and was based on a high-pressure blower, two pressure transducers, and an Arduino Nano controller with a digital display (total retail cost < 75 USD$). The ventilator was first evaluated and compared with a commercially available device (Lumis-150, Resmed) on the bench using an actively breathing patient simulator mimicking a range of obstructive/restrictive diseases. The low-cost ventilator was able to provide inspiratory/expiratory pressures of up to 20/10 cmH_2_O, respectively, with no faulty triggering or cycling. The ventilator was also tested in 12 healthy volunteers wearing a high airway resistance and thoracic/abdominal bands to mimic obstructive/restrictive patients. When applied under conditions mimicking patients, the ventilator was able to support the subjects’ breathing with highly demanding inspiratory and expiratory pressures with no artifacts in cycling and triggering. Figure 5B shows an example of the pressure signal (provided by the ventilator sensor) when a volunteer mimicking an obstructive–restrictive patient was supported with strenuous inspiratory and expiratory pressures of 22 and 10 cm H_2_O, respectively. Interestingly, Figure 6 shows that the ventilator function was comfortable for subjects. Indeed, the breathing difficulty score rated (1–10 scale) by the loaded breathing subjects was significantly (*p* < 0.005) decreased from 5.45 ± 1.68 without support to 2.83 ± 1.66 when using the prototype ventilator, which showed no difference compared with the commercial device (2.80 ± 1.48; *p* = 1.000). Consequently, this open-source proposal for a low-cost, easy-to-build, non-invasive ventilator performs similarly to a high-quality commercial device, thereby allowing for free replication and use in LMICs, and thus, facilitating the application of this life-saving therapy to patients who otherwise could not be treated.

## 4. Discussion

The examples of low-cost (all of them below 100 €) and simple devices shown in the previous section illustrate how high-performance respiratory medical devices can be designed and built following a free open-source approach. It is interesting to note that many respiratory devices, as well as many devices in other medical specialties, are particularly suitable for this production approach because of their technical simplicity. Indeed, they are based on principles and methods developed several years, and even decades, ago. In addition, most of these devices can be built by small teams, e.g., within University laboratories or public hospitals units, not requiring large industrial infrastructures and companies (as would be the case to fabricate CAT scanners or MRI equipment; although, it should be pointed out there are open-source efforts on these fronts as well, e.g., https://www.opensourceimaging.org/, accessed on 13 August 2022). For instance, the example devices presented above were developed and constructed within the framework of the final degree theses of biomedical engineering students. Whereas the know-how required to achieve the design and to test the device performance required engineers and doctors with great experience in technology and respiratory medicine, replication of the devices using the open-source information provided can be carried out by anyone having trained at the level of last-year engineering degrees with minimal electronic workshop tools and now common desktop 3D printers, which makes it feasible in LMICs. In this context, it is worth mentioning that the implementation and generalized use of a considerable number of published medical device solutions that are low-cost but are not open-access would be greatly enhanced should an open-source version become available.

Designing and distributing open-source projects for medical devices is so simple that two cautionary notes should be issued. First, there is a plethora of such projects in the literature, particularly in technological journals and environments, but very few of them have been carried out by teams that not only develop a technical solution but also, and most importantly, involve expert healthcare professionals. Ideally, medical experts are involved in the design and the evaluation of the devices, first on the bench and subsequently in patients. For example, during the first two years of the COVID-19 pandemics, when the lack of available mechanical ventilators was an important problem worldwide [63,64], a considerable number of low-cost, simple ventilator projects were published [58]; however, most of these projects presented the technical design with no or only with minor realistic testing in patient models, and only an exceptional minority were tested in vivo either in realistic animal models (e.g., porcine acute lung injury) or in patients. These devices provided a good open-source foundation for developing fully functional and regulated ventilators but needed additional work to make them available for anything other than an emergency. Noteworthily, open-source medical devices with the potential for translation to clinical application should be thoroughly prepared, going far beyond the relatively simple initial stage of technological design, involving a team with wide expertise including electronic and instrumentation design experts, specialists in respiratory physiology, and clinical practitioners. Specifically, open-source routines and circuits should be tested under well-controlled conditions simulating real-life clinical scenarios, and the documentation describing the devices should clearly explain the exact conditions and operating procedures of their intended use. Moreover, if the project is aimed at development in LMICs, it is important to also involve local professionals to ensure that the project is suitable for implementation under local conditions [65,66]—specifically, to facilitate “personalizing” the project to the characteristics of on-site healthcare staff and patients. Therefore, completing such an open-source project is not an easy task for an individual or even an established research group. Using the open-source model, however, many people with the requisite skills can collaborate building the necessary expertise as a whole. What should be very easy is the fabrication of the devices by potential users by following the clear and detailed technical descriptions provided by the project authors that are readily available online. A second cautionary note regarding the ease of obtaining the information to build open-source devices is that well-meaning people without adequate technical/biomedical training may dangerously misuse it. It is fundamental to discourage such practice while further including cautionary statements that inform a priori the dangers associated with such misinformed initiatives.

The open-source approach for fabricating medical devices in LMICs is a path clearly different from the conventional proprietary-based system of products mainly commercialized by companies in developed countries. The aim is not necessarily trying to establish a commercial competence simply because, in practice and for financial reasons, the commercial market is not operating in LMICs (with the exception of providing products for a small minority of wealthy patients). This situation is not expected to change in the upcoming decades given the huge gap between costs and prices in developed countries and financial capabilities in LMICs. In this context, the open-source approach appears as a possible route enabling access to medical devices for use by a significant proportion of patients in low-resource regions, and this approach is already seeing results in closing the gap between the haves and have-nots in scientific instrumentation [67]. There is room to define what could be the best model to implement the open-source approach. Probably, different solutions (e.g., commercial using open-source business models [68,69], or voluntary production [70]) could be implemented depending on the specific characteristics of each LMIC. A promising possible model to start developing the production is such that the low-cost medical devices are built by small technical teams linked to hospitals or to associated engineering schools, which could work independently or be coordinated at the regional or national levels. This would, for instance, mimic the successful process of building ventilators, CPAP devices, and other innovations in the early times of invention and development of these devices [71].

A fundamental issue to be addressed regarding the local fabrication of medical devices is their safety and patient protection. The normal rule in developed countries is that any medical device to be used on patients should have been approved by the corresponding regulatory body (e.g., FDA, CE mark). This process has the advantage of ensuring, at a national or international level, that a device complies with minimal quality and safety standards and conditions. These regulatory agencies were created to protect patients (and clinicians) from using low-quality or unsafe medical devices. This protection model certainly works well in the market of developed countries. Unfortunately, the complexity and cost of obtaining regulatory approval has morphed into a barrier against innovation and competition from new companies entering the medical technology market [72,73,74]. Indeed, the whole process required to obtain approval by any of these agencies is often prohibitively expensive (and time-consuming), even for companies in those countries. Obviously, this model for medical device safety control is conceptually and practically not applicable for devices locally fabricated by the open-source approach. Although it may appear in developed countries that control by national regulatory agencies is the only possible procedure for ensuring safety and patient protection, it is not and was not the only possibility. Indeed, decades ago in developed countries, when most innovations on medical devices were invented and developed by building them locally before being industrialized, patient safety was ensured by inspection and approval by hospital ethical boards [71], which in fact is the way for currently approving clinical trials [75,76]. This local model was in the past, and continues nowadays, to be useful for protecting patient safety when subjected to new medical procedures. Therefore, this process could be equally applied to approving devices built locally, provided that the ethics committees are supported by technical professionals, either by including them in the ethical boards or by establishing procedures for external consultation with technical experts prior to ethical board approval. By sharing the requirements, using this method would ensure patient safety while leveraging the benefits of rapid innovation and low costs observed with the open-source approach. Establishing the concept that, to be used in LMICs, medical devices must have an FDA or CE mark is in practice equivalent to prohibiting most inhabitants from having access to potentially life-saving treatments and makes extremely difficult the development of a local industry of medical devices.

In addition, developed economy medical regulators could also learn from the open-source approach, as currently there are major challenges to using distributed manufacturing of open medical hardware in regulated areas. For example, for those operating in the U.S., for open hardware to be used clinically an Investigational Device Exemption (IDE) is needed to allow for a non-FDA-approved device. This is only temporary, and the complete device would need full FDA approval for legal deployment (with the exception of when laws are temporarily changed or suspended during a pandemic [77]). Emergency use authorization (EUA) was approved for a number of technologies [78] during the pandemic for those both made in the U.S. and imported [79]. Regulations for specific classes of medical technology could improve innovation rates by having the technical validation/testing required published openly so that developers could ensure their technologies meets requirements before anyone attempts to obtain regulatory approval. Having clear, freely available methods of validation and testing allows for rapid response and deployment of needed medical supplies and technologies during a pandemic.

## 5. Conclusions

As summarized in Table 1, the relatively novel, free open-source approach for design and distribution may be a uniquely useful and valuable tool for facilitating affordable respiratory medical devices for patients in LMICs who otherwise would not have access to the instruments and devices that, in some cases, enable life-saving diagnosis and treatment. The common practice of approving clinical trials by the local or hospital ethics board can be expanded to ensure patient safety by reviewing, inspecting, and approving open hardware for medical applications [80]. The sharing of the tests required can be made openly accessible to maximize the innovation and deployment rate of medical technologies.

## Figures and Tables

**Table 1:** Summary of the main features of medical devices from the conventional industry market and from open-source local production.

Item	Sub-Item	Conventional Industry Market	Open-Source Local Production
Technological complexity of the device	Very high	YES	DIFFICULT *
	Low–medium	YES	YES
Cost and availability for most patients	Cost	EXPENSIVE	CHEAP
	Availability	LOW	HIGH
Health provider perspective	Local servicing/repair	DIFFICULT	EASY
	Adaptability to local needs	DIFFICULT	EASY
	Local industry promotion	LOW	HIGH
	Requiring team-building initiative	NO	YES
Regulations and safety requirements	International standards (FDA, CE)	YES	DIFFICULT
	Local approval	NO	YES

* Difficult with current distributed manufacturing technologies. However, it should be noted that with coming open-source electronics and other multimaterial 3D printing, micromachining, and similar technologies, the current barrier to very-high-complexity devices will be overcome.

## Data Availability

Not applicable.
